# Fabrication of conductive silver paste recovered from leaching of waste catalyst using hydrochloric acid

**DOI:** 10.1039/d1ra09435a

**Published:** 2022-03-28

**Authors:** Suhyeon Lee, Brakowaa Frimpong, Stanley Abbey, Yoon Sil Moon, Kyoungkeun Yoo, Young-Min Oh, Soo-Kyung Kim, Sang-Joon Kim, Min-Wook Oh

**Affiliations:** Department of Advanced Materials & Chemical Engineering, University of Science & Technology Daejeon Republic of Korea; Department of Materials Science and Engineering, Hanbat National University Daejeon Republic of Korea mwoh@hanbat.ac.kr; Mineral Resources Research Division, Korea Institute of Geoscience & Mineral Resources Daejeon Republic of Korea; Department of Energy & Resources Engineering, Korea Maritime and Ocean University Busan Republic of Korea; Danam-ene, Co. Gyeonggi-do Republic of Korea; Environment and Sustainable Resources Research Center, Korea Research Institute of Chemical Technology Daejeon Republic of Korea

## Abstract

Transition metal compounds based on silver (Ag) and palladium (Pd) are extensively used as catalysts in the petrochemical industries. The catalytic activities of Ag and Pd decrease over time and hence need to be discarded. The recovery of elements like Ag from waste catalyst is essential because of its limited availability and cost, and it is environmentally beneficial with regards to recycling. In this study, Pd and Ag were leached from waste catalyst providing an alternative source suitable for a Ag paste electrode. Through an efficient reduction process, AgCl particles were obtained which serve as a precursor to synthesize Ag using ammonia as the solvent. The obtained Ag was fabricated to Ag paste by using mixed dispersion and solvent. The electrical resistivity of the Ag paste was recorded as 6.14 μΩ cm at 417 °C in a hydrogen atmosphere.

## Abstract 2

The silver paste was synthesized by a hydrometallurgical method from waste catalyst.

## Graphical abstract

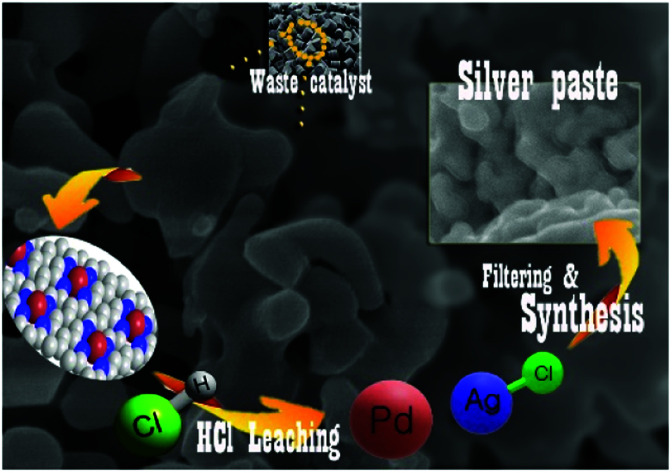

## 1. Introduction

Catalysis involves the process of increasing the rate of a chemical reaction. Catalysts are particularly attractive materials that speed up a chemical reaction by decreasing the activation energy barrier. Catalysts are utilized by various industries to make petrochemical feedstock which are the basic raw materials for manufacturing plastics, synthetic rubber and polyester fibre.^1–3^ The catalytic materials mainly contain transition group elements, such as palladium (Pd), platinum (Pt) and rhodium (Rh).^4–6^ Palladium and silver catalysts are widely used in petrochemical processes such as carbon bond-forming reactions.^7–10^ Pd and Ag are precious metals (PMs), rare and very expensive.^11^ Ag is usually used as an electrode for devices due to its high corrosion resistance and electrical conductivity. Liu *et al.*^12^ fabricated silver paste that has an electrical resistivity of 3.31 ± 0.73 × 10^−5^ Ω cm at 200 °C for 45 min in air. Currently, several scientific studies are underway to recover these metals by absorption from waste catalyst using simple but effective leaching methods.^13–15^ In terms of the green chemistry, the primary goals are the minimization of the waste catalysts and recovering PMs. Among various methods to recover PMs, the hydrometallurgical method is one of the conventional ways for Pd, Au and Ag. The method typically involves dissolution of metals and reduction of ionized metals from the leachate. There are many leaching agents such as aqua regia, HNO_3_, HCl, thiourea, and thiosulfate. However, it has been proved that each leaching agent is effective for only one or two PMs.^16,17^ Therefore, it is needed to improve the performance of the leaching agent.

Recently, AgCl has been successfully retrieved from electronic scrap and used for the synthesis of silver nanoparticles.^18^ In Ag nanoparticles synthesis, AgCl can be initially formed by addition of Cl^−^ precursors to AgNO_3_ salt serving as a nucleant for the growth. AgCl formation results in the stabilizing of the Ag nuclei to prevent agglomeration by slowing down the reduction of Ag^+^ to Ag^0^. The AgCl formed can then be slowly reduced to control nanoparticle size.^19–21^ AgCl synthesized from waste catalyst could be a great alternative for this purpose.

It is generally known that Pd can be leached with HCl or aqua regia and Ag with HNO_3_. In this study, it was found that HCl can be utilized to leach both Pd and Ag. Pd was successfully leached and AgCl was formed by utilization of HCl. And Ag was obtained by subsequent dissolution of AgCl by addition of ammonia water and a reducing agent, NaBH_4_. Even though the leaching efficiency of HCl for Ag is low due to low content of Ag and preferred dissolution of Pd compared with Ag, the finding will shed light on further research into efficient leaching agents. The obtained Ag particles were synthesized into Ag paste using a dispersant and solvent. The shape of the synthesized Ag particles was characterized using SEM. The compositions of the reduced metal particles from the leachate and the synthesized Ag particles were examined by EDS. The XRD results confirm the crystallinity of the reduced metal particles from the leachate and the synthesized Ag particles. Silver film was fabricated by tape casting in order to measure the electrical resistivity of the silver paste by Hall measurements.

## 2. Experimental details

### 2.1 Waste catalyst leaching

**Fig. 1:**
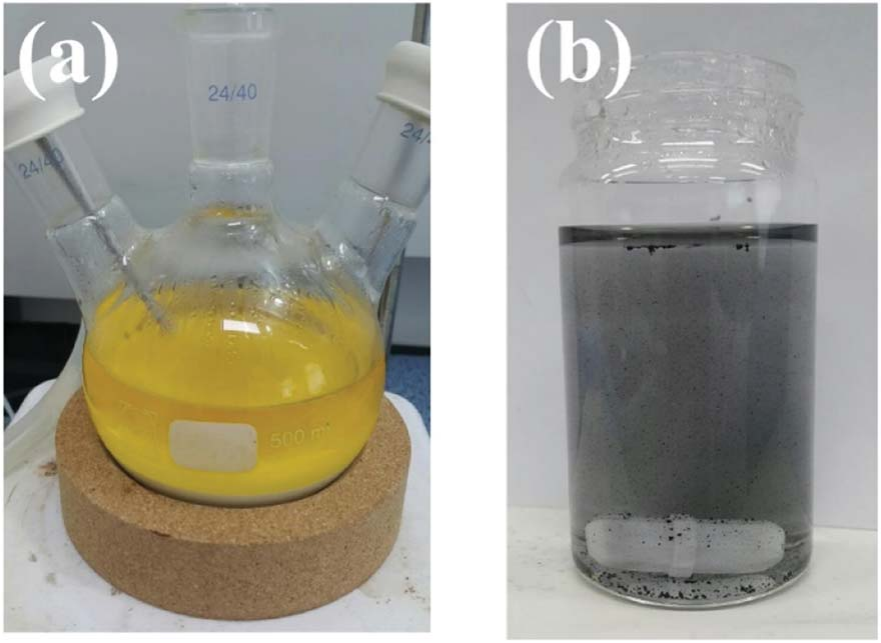
Pictures: (a) waste catalyst and leachate after leaching process and (b) reduced leachate using NaBH_4_ reductant.

The waste catalyst of size 4.5 mm is pulverized with a roll milling machine and ground to ∼100 μm using an agate mortar and pestle. 50 g of the pulverized waste catalyst is loaded into a three-neck round bottom flask with 300 ml of 4 M HCl solution (28%, Junsei) and then stirred at 700 rpm using a magnetic bar. The temperature of the solution is maintained at 90 °C for 1 h using a heating mantle. After cooling to room temperature, qualitative filter papers are used to separate the waste catalyst residue and leachate. In order to ascertain the exact amount of leachate that has been reduced by filtration of waste catalyst residue, the process is repeated consecutively six times by adding a solution of 100 ml of 4 M hydrochloric acid to the leachate and waste catalyst. 300 ml of the leachate is then mixed with 10 ml of deionized (DI) water and 1 M NaBH_4_ using a magnetic bar for 10 min in a syringe to reduce the metals ionized in the leachate. The reduced metal particles were analysed after drying. Fig. 1 shows pictures of the waste catalyst, leachate, and reduced leachate.

### 2.2 Ag particles synthesized from AgCl precursor

Ag particles were synthesized using AgCl (reagent grade, 99.9%, Alfa Aesar) and Pd (powder, <1 μm, ≥99.9%, Sigma-Aldrich). Ammonia water (NH_3_·H_2_O, 28%, Junsei) was used to dissolve the AgCl precursor. Trisodium citrate dihydrate (99%, Sigma Aldrich) was used as a surfactant. Sodium borohydride (NaBH_4_, 98%, Sigma Aldrich) was used as a reducing agent. 200 ml of 3 M ammonia water was added to 0.1 M AgCl and 0.1 M Pd and then stirred at 400 rpm using a magnetic bar for 20 min. After visually checking that the AgCl precursor is clearly dissolved, the leachate was separated into Pd powder and ionized AgCl using syringe filters. After that, 0.01 M trisodium citrate dihydrate is added and continuously stirred for 10 min. 1 M NaBH_4_ in 10 ml of DI-water was stirred at 400 rpm for 10 min using a magnetic bar. The contents of a syringe in which the surfactant and the precursor were dissolved were then added to the ammonia water. After further stirring for 30 min, the solution was centrifuged at 1000 rpm for 10 min and then washed twice with ethanol.

The obtained Ag particles, polypropylene glycol and BYK-425 dispersant are measured out in a weight ratio of 1 : 0.4 : 0.1, and then mixed by ultra-sonication to form a paste. The Ag films were fabricated by depositing Ag paste on a SiO_2_ wafer by tape-casting and then annealed using a tube-furnace in a hydrogen atmosphere to prevent oxidation.

### 2.3 Evaluation method for particles and film

The composition and amount of waste catalyst were measured using an Inductively Coupled Plasma Mass Spectrometer (Agilent ICP-MS 7700S, Agilent technologies, Inc., American). The shape of the metal particles and the thickness of the films obtained from the waste catalyst and the wet metallurgical method were recorded using a field emission scanning electron microscope (FE-SEM S-4800, Hitachi, Ltd., Japan). The compositional and structural analysis was performed using energy-dispersive X-ray spectroscopy (EDS, X-MaxN, Oxford Instruments, Abingdon, UK) and an X-ray diffractometer (SmartLab, Rigaku Co., Japan). The electrical properties were measured by a Hall effect meter (Ecopia HMS-5000, Four-point probe, Republic of Korea). All the measurements were carried out under ambient air.

## 3. Results and discussion

### 3.1 Waste catalyst ingredients analysis

**Fig. 2:**
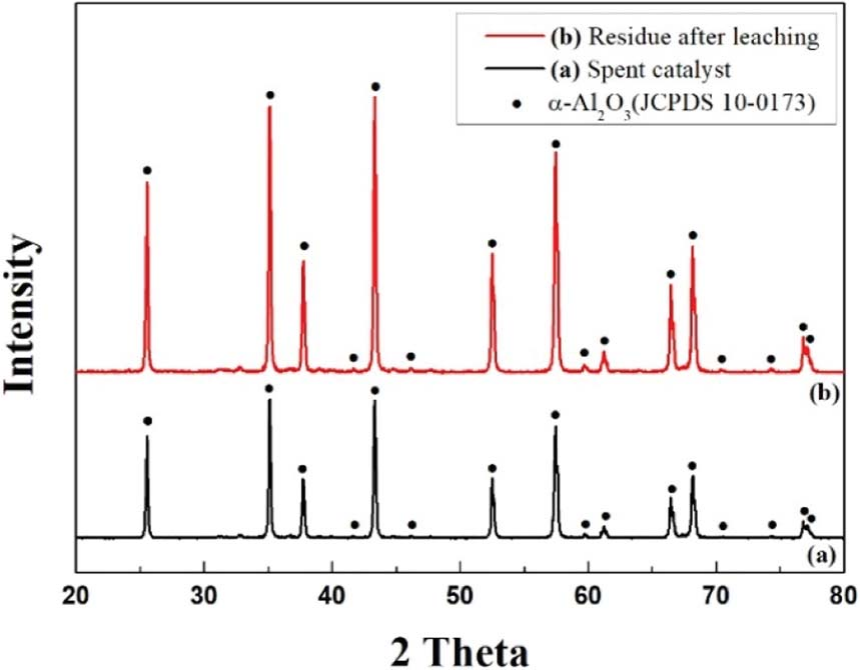
XRD patterns: (a) pulverized waste catalyst and (b) residue after leaching the pulverized waste catalyst with HCl solution.

XRD measurements were performed to identify the phase of the waste catalyst powder. Fig. 2 shows the XRD results of pulverized waste catalyst and residues after leaching with HCl solution. The XRD diffraction peaks are indexed to the α-Al_2_O_3_ phase which has a hexagonal *R*3̄*c* lattice structure when compared with Joint Committee on Powder Diffraction Standards (JCPDS) with PDF number 10-0173. Only α-Al_2_O_3_ phases were observed in the residues after leaching with HCl. This is attributed to the dispersion of the waste catalyst into the porous α-Al_2_O_3_ particles. No other phases such as Pd and Ag were detected due to the small amounts of them, which are below the limits of resolution of the XRD.

**Fig. 3:**
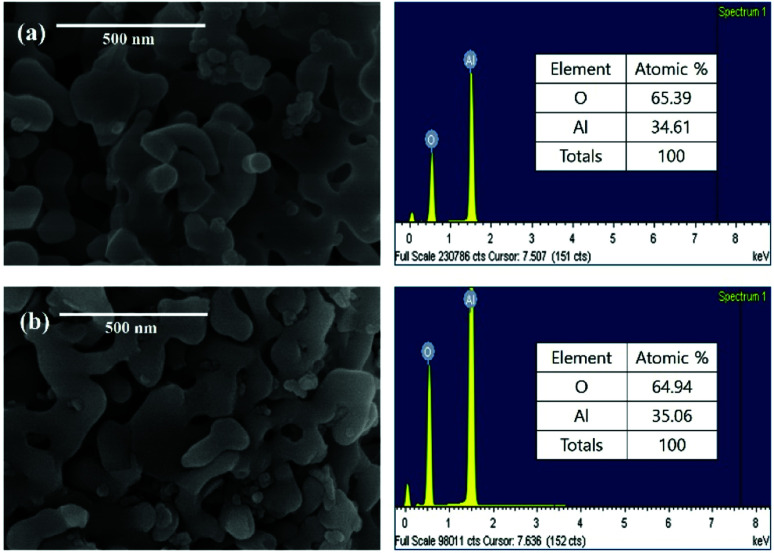
SEM and EDS results: (a) powdered waste catalyst and (b) residue after leaching the ground waste catalyst with HCl solution.

Fig. 3 shows the SEM and EDS of powdered waste catalysts and residue after leaching the waste catalyst with HCl solution. The SEM results confirm that shape and size of α-Al_2_O_3_ particles are not changing before and after leaching of the catalytic metals. The corresponding EDS results confirms the existence of α-Al_2_O_3_ particles.

ICP-MS was used to determine the concentration of metals absorbed in the waste catalyst. Table 1 shows the results of the ICP-MS of waste catalyst leached with HCl solution. Pre-treatment of the waste catalyst with 4 M HCl solution at 90 °C for 1 h was used to measure the ionizing metals that act as a catalyst. ICP-MS results showed that the metals used as a catalyst contained platinum group metals in the decreasing order of Al > Pd > Ag. Al in the leachate originated from the Al_2_O_3_ support, and Pd and Ag in the leachate come from the catalytic elements.

**Table 1:** ICP-MS results for the leachate

Metals	ppm (mg kg^−1^)
Al	851.496
Pd	52.840
Ag	26.099

The metals detected using ICP-MS react with HCl as shown in the following reactions eqn (1)–(3).^22–28^

1 Al_2_O_3_ + 8HCl → 2AlCl_4_^−^ + 3H_2_O + 2H^+^

2 Pd + 4HCl → [PdCl_4_]^2−^ + H_2_ + 2H^+^

3 2Ag + 4HCl → 2[AgCl_2_]^−^ + H_2_ + 2H^+^

Waste catalysts containing Al, Pd, and Ag metals were leached with HCl solution. The ionized metals were reduced by adding a reducing agent and then analysed.

### 3.2 Analysis of reduced metal particles leached from waste catalysts using HCl solvent

**Fig. 4:**
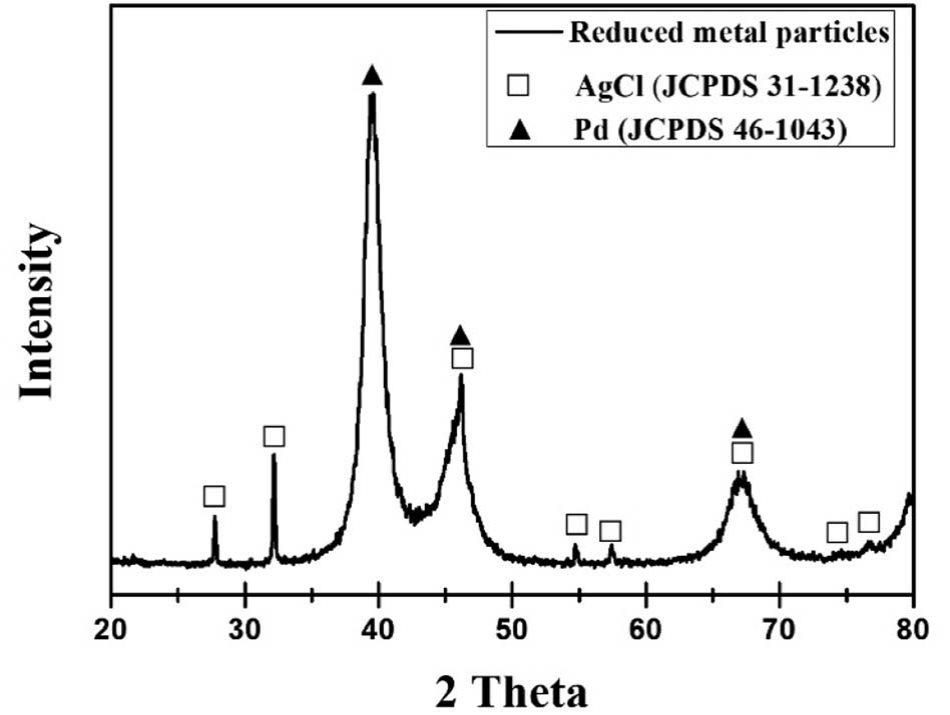
XRD pattern of reduced metal particles from leachate.

**Fig. 5:**
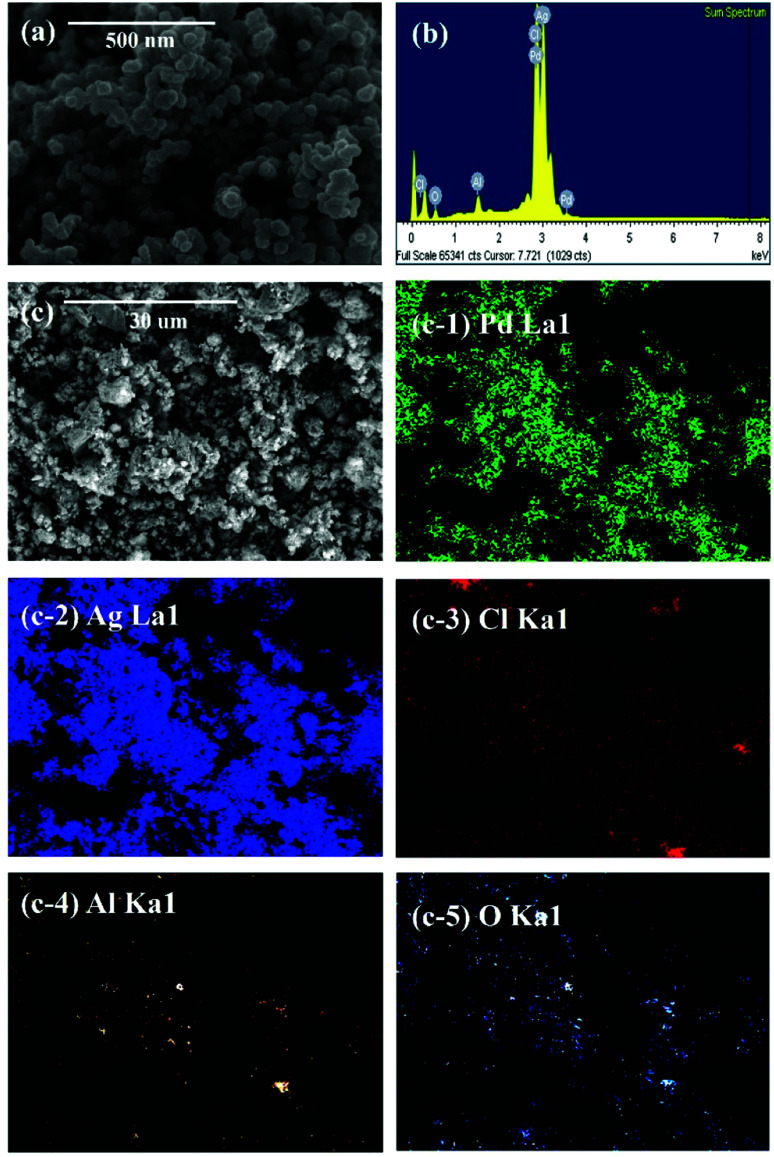
SEM, EDS, and EDS mapping of particles reduced from leachate: (a) SEM image, (b) EDS result, (c and c-1–5) SEM image for EDS mapping and EDS mapping results.

Fig. 4 shows the XRD pattern of the reduced particles from the leachate. Peak positions at 40.118°, 46.658°, and 68.119° correspond to the (111), (200) and (220) planes of Pd, respectively. Also, other diffraction peaks are identified as a phase of AgCl (JCPDS 31-1238) with the main peak at 32.243°. Fig. 5(b) depicts EDS results, confirming that the peaks observed in XRD are of Pd and AgCl. No reduction of Al is due to the fact that aluminium has a small standard electrode potential (*E*^0^(V) = −1.676) compared to other metals.^29^

Fig. 5 shows the results of SEM and EDS mapping of particles reduced from leachate. Fig. 5(a) and (c) show the SEM images indicating the shape of the reduced particles. Fig. 5(b) and (c-1–5) are EDS mapping results. The results of XRD and EDS mapping show that Pd, AgCl, and Al_2_O_3_ were detected. Aluminium is not reduced in the chemical process, however, EDS mapping indicated the presence of Al_2_O_3_. This is due to the permeation of Al_2_O_3_ during the filtration of the leached waste catalyst using qualitative filter papers. Because of its small distribution in the form of nanoparticles, it was not observed in the XRD results but Al_2_O_3_ was detected by EDS mapping results.

Addition of NaBH_4_ results in the reduction of Pd metal to Pd instead of PdCl_2_. [PdCl_4_]^2−^ was present in the form of a solution and is not reduced to solid metal particles.^30^ However, AgCl_2_^−^ was reduced to AgCl with no evidence of Ag particles.

Eqn (4) and (5) are reactions of NaBH_4_ as a reducing agent with an ionized material.^31^

4 [PdCl_4_]^2−^ + NaBH_4_ + 4H_2_O → Pd + NaB(OH)_4_ + 4Cl^−^ + 3H_2_ + 2H^+^

5 2[AgCl_2_]^−^ + NaBH_4_ + 4H_2_O → 2(AgCl) + NaB(OH)_4_ + 2Cl^−^ + 4H_2_

The chemical reduction processes above resulted in reduction to Pd and AgCl. This confirms that the leaching process for the waste catalyst was successful.

### 3.3 Highly conductive Ag paste fabricated using AgCl precursor

**Fig. 6:**
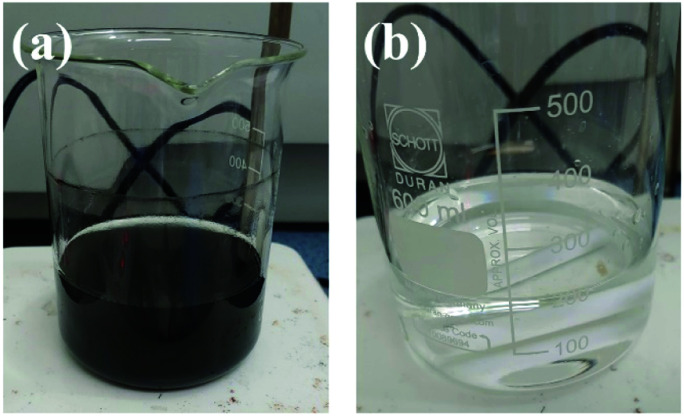
Pictures of the leachate: (a) Pd and AgCl precursor in ammonia solvent, (b) leachate after filtering by syringe filter.

To enhance the added value of the recovered silver particles, we prepared Ag paste from the recovered silver particles and measured the electrical resistivity to estimate a key property of the paste. A minimum of 1 g of Ag particles is required from the waste catalyst to synthesize Ag paste. The ICP-MS results indicate that Ag is ionized to about 26.099 mg kg^−1^. The calculated results show that 38.31 kg of leached waste catalyst is required to produce Ag paste. It is believed that there are two reasons for the low leaching efficiency of Ag from the waste catalysts. Firstly, we recovered Ag particles from waste catalysts produced at a petrochemical company in Korea. The waste catalysts contained both Pd and Ag supported by Al_2_O_3_ (Pd–Ag/Al_2_O_3_), in which the amount of Pd is larger than that of Ag. However, the conventional Ag/Al_2_O_3_ catalysts used in fabricating formaldehyde from methanol and ethylene from ethylene oxide contain 8–20 wt% of Ag.^8^ Therefore, the content of Ag in our waste catalysts is much smaller than the conventional Ag/Al_2_O_3_ catalysts. Secondly, HNO_3_ is generally used as a leaching agent to recover Ag from the waste Ag/Al_2_O_3_ catalysts, while HCl and aqua regia are usually used to recover Pd from Pd/Al_2_O_3_ catalysts.^16^ We aimed to recover both Pd and Ag with HCl from the Pd–Ag/Al_2_O_3_ catalysts. Therefore, it is believed that the low leaching efficiency is attributed to the low content of Ag in the waste catalysts and utilization of HCl as the leaching agent for both Pd and Ag. As such it is difficult to use AgCl reduced from the leachate. Ag particles were synthesized using reagent grade AgCl and Pd precursors. Ag paste with high conductivity was produced by dissolving AgCl metal particles in ammonia water solvent, using NaBH_4_ as a reducing agent. Ammonia water (NH_3_·H_2_O) was used as a solvent to ionize AgCl. Pd dissolved very slowly at room temperature in NH_3_·H_2_O solvent.^32–35^ Pd and AgCl can be separated from reduced metal powder by the application of syringe filters. Fig. 6 shows evidence of leachate containing Pd and AgCl in ammonia solvent and the final solution after separation with syringe filters.

Eqn (6) and (7) are reaction formulae of AgCl and NH_3_·H_2_O and Ag reduction with NaBH_4_.

6 AgCl + 2(NH_3_·H_2_O) → [Ag(NH_3_)_2_]^+^ + Cl^−^ + 2H_2_O

7 2[Ag(NH_3_)_2_]^+^ + 4NaBH_4_ + 2Cl^−^ + 4H_2_O → 2Ag + 4NaNH_2_ + 4B(OH_3_) + 2HCl + 7H_2_

**Fig. 7:**
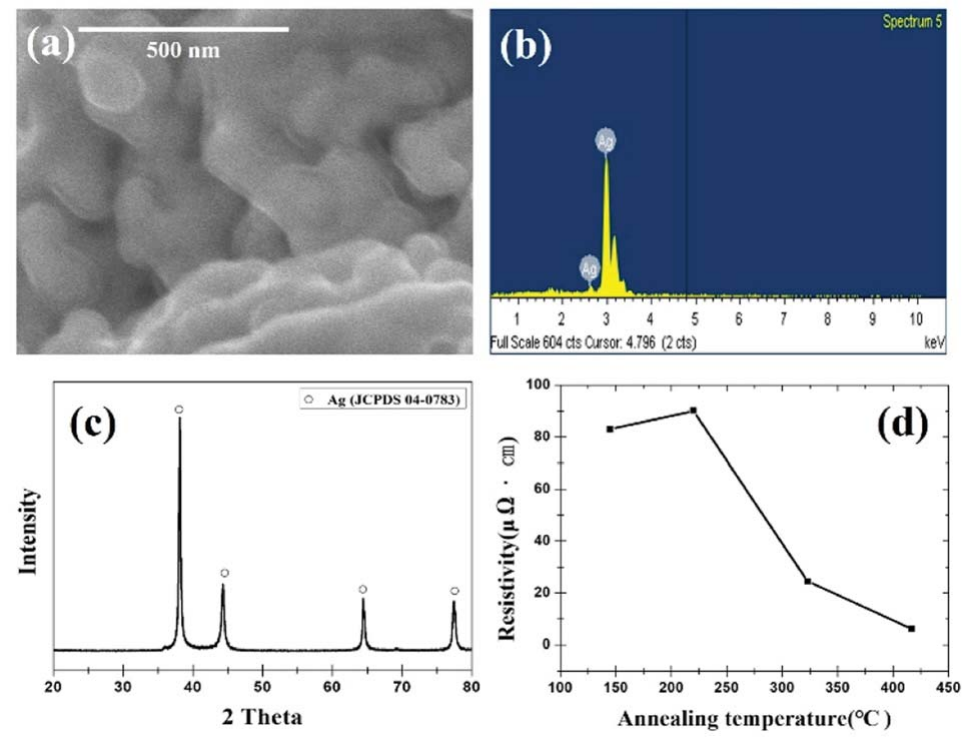
Synthesized Ag nanoparticles: (a) SEM image, (b) EDS result, (c) XRD pattern, and (d) temperature-dependent resistivity of Ag paste.

Fig. 7 shows an SEM image, EDS results, XRD pattern, and resistivity of Ag nanoparticles synthesized from the AgCl precursor. The SEM image in Fig. 7(a) is not clear because Ag nanoparticles were surrounded by the trisodium citrate as capping agent. EDS and XRD results in Fig. 7(b and c) show the successful synthesis of Ag nanoparticles from AgCl. The resistivity of the Ag paste reduces as the heat-treatment temperature increases, as shown in Fig. 7(d). The annealing of the samples was performed at setting temperatures of 300 °C, 400 °C, 500 °C and 600 °C, respectively. However, the real temperatures of the samples were measured as 145 °C, 220 °C, 323 °C and 417 °C, respectively, using a thermocouple. The resistivity values of the synthesized silver films are 82.99 μΩ cm (145 °C), 90.09 μΩ cm (220 °C), 24.42 μΩ cm (323 °C), and 6.14 μΩ cm (417 °C) in a hydrogen atmosphere. The lowest value of the resistivity was comparable to the literature in which the electrically conductive silver paste was studied.^12,36,37^ It is believed that electrically conductive paste can be one of the applications for the recovered Ag particles.

## 4. Conclusions

Waste catalyst is composed of α-Al_2_O_3_ support, Pd, and Ag. Pd and Ag can be leached using a hydrochloric acid solution. The EDS mapping and XRD results confirm that AgCl and Pd were leached from waste catalysts by HCl solution. AgCl was ionized using NH_3_·H_2_O solvent in order to fabricate silver paste. Ag nanoparticles were synthesized by using trisodium citrate as surfactant, and NaBH_4_ as a reducing agent. Ag paste was prepared by adding dispersant and solvent to the silver nanoparticles and analysed after annealing. The Ag paste showed a resistivity of 82.99 μΩ cm (145 °C), 90.09 μΩ cm (220 °C), 24.42 μΩ cm (323 °C), and 6.14 μΩ cm (417 °C), respectively.

## Conflicts of interest

There are no conflicts to declare.

## Supplementary Material
